# Photoinduced Energy Transfer from Poly(*N*-vinylcarbazole) to Tricarbonylchloro-(2,2'-bipyridyl)rhenium(I)

**DOI:** 10.1002/cphc.201402269

**Published:** 2014-08-19

**Authors:** Engelbert Portenkirchner, Dogukan Apaydin, Gottfried Aufischer, Marek Havlicek, Matthew White, Markus Clark Scharber, Niyazi Serdar Sariciftci

**Affiliations:** [a] Linz Institute for Organic Solar Cells (LIOS), Physical Chemistry, Johannes Kepler University Linz (Austria) E-mail: engelbert.portenkirchner@jku.at

**Keywords:** catalysis, CO_2_ reduction, energy transfer, poly(*N*-vinylcarbazole), rhenium

## Abstract

This work investigates the photoinduced energy transfer from poly(*N*-vinylcarbazole) (PVK), as a donor material, to *fac*-(2,2'-bipyridyl)Re(CO)_3_Cl, as a catalyst acceptor, for its potential application towards CO_2_ reduction. Photoluminescence quenching experiments reveal dynamic quenching through resonance energy transfer in solid donor/acceptor mixtures and in solid/liquid systems. The bimolecular reaction rate constant at solution–film interfaces for the elementary reaction of the excited state with the quencher material could be determined as 8.8(±1.4)×10^11^ L mol^−1^ s^−1^ by using Stern–Volmer analysis. This work shows that PVK is an effective and cheap absorber material that can act efficiently as a redox photosensitizer in combination with *fac*-(2,2'-bipyridyl)Re(CO)_3_Cl as a catalyst acceptor, which might lead to possible applications in photocatalytic CO_2_ reduction.

## 1. Introduction

The global energy supply depends on limited fossil-fuel resources that produce substantial amounts of the greenhouse gas carbon dioxide upon combustion. This problem has created significant scientific interest in the capture and utilization of carbon dioxide.[1–3] An encouraging approach is the reduction of atmospheric carbon dioxide to synthetic carbon-neutral fuels with the use of solar energy as an abundant and renewable energy resource.[4–6]

*fac*-(2,2'-Bipyridyl)Re(CO)_3_Cl [(2,2'-bipy)Re(CO)_3_Cl] is known to be an efficient photocatalyst for the reduction of CO_2_, producing mainly CO, with the aid of a sacrificial electron donor such as triethanolamine (TEOA). The quantum yield (*Φ*_CO_) for photocatalytic CO_2_ reduction with this compound was reported as 0.14 almost 30 years ago by Lehn et al. in 1984.[7, 8] Subsequent modifications of the bipyridine ligand system of (2,2'-bipy)Re(CO)_3_Cl have led to the development of superior catalysts with *Φ*_CO_ values up to 0.59, making this type of material the most efficient CO_2_ photocatalyst, to date, among known homogeneous catalyst materials.[9, 10]

Unfortunately, rhenium tricarbonyl complexes have several disadvantages when it comes to practical applications, with their poor absorption of the solar spectra [molar absorption coefficient at 340 nm (*ε*_340_) of 1.5(±0.2)×10^5^ m^−1^ m^−1^, compare Figures S1 with S2 a and b in the Supporting Information] and the low abundance of rhenium as the most prominent drawbacks.[11] One promising solution to overcome the disadvantages of rhenium-based catalysts is to use poly(*N*-vinylcarbazole) (PVK) as a good and cheap absorber (*ε*_340_ 2×10^9^ m^−1^ m^−1^)[12] to serve as redox a photosensitizer in combination with (2,2'-bipy)- Re(CO)_3_Cl as a catalyst acceptor.

**Scheme 1:**
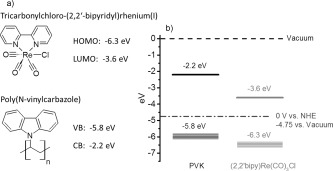
a) Chemical structure of (2,2'-bipy)Re(CO)_3_Cl with its corresponding HOMO and LUMO levels (in eV), and PVK with its corresponding energy values for VB and CB. b) Schematic drawing of the frontier orbital energy levels, comparing PVK as the donor and (2,2'-bipy)Re(CO)_3_Cl as the acceptor molecule.

Scheme 1 a shows the chemical structure of (2,2'-bipy)Re(CO)_3_Cl and its corresponding highest occupied molecular orbital (HOMO) and lowest unoccupied molecular orbital (LUMO) level, determined to be −6.3 eV versus vacuum for the HOMO and −3.6 eV versus vacuum for the LUMO. The values were determined from electrochemical and UV/Vis absorption measurements, as well as quantum mechanical calculations on the DFT level (compare Figures S1, S3, and S4 in the Supporting Information). Additionally, Scheme 1 a shows the chemical structure of PVK and its corresponding energy values for the valence band (VB) and the conduction band (CB) as −5.8 and −2.2 eV versus vacuum, respectively, which were taken from literature values.[13] Scheme 1 b shows the schematic drawing of the material frontier orbital energy levels comparing PVK as a donor and (2,2'-bipy)Re(CO)_3_Cl as an acceptor molecule versus the vacuum energy. For the electrochemical consideration of frontier orbital energy levels, an offset of the normal hydrogen electrode (NHE) potential versus the vacuum level of −4.75 eV is used.[14] As can be seen in Scheme 1 b, the energy levels of the donor polymer and acceptor catalyst are aligned in a favorable situation for photoexcited charge and/or energy transfer. Subsequent photoluminescence (PL) quenching and light-induced electron spin resonance (ESR) experiments were carried out to study this system in bulk, in solid phase mixtures of donor and acceptor, and at a donor–acceptor solid–liquid interface.

## 2. Results and Discussion

### 2.1. PL Quenching in Solid Films

**Figure 1:**
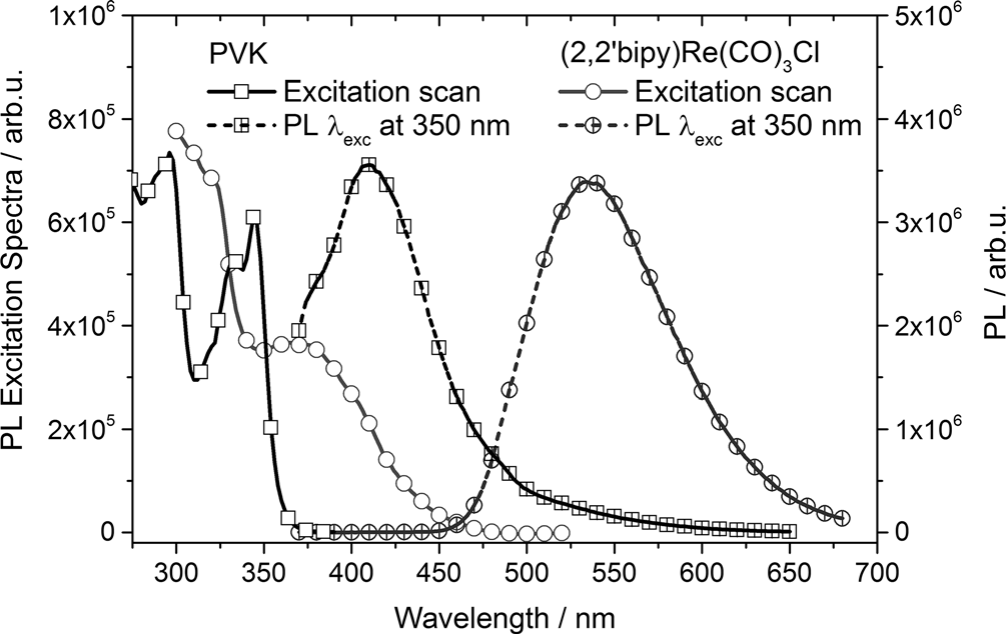
Excitation and PL spectra of PVK and (2,2'-bipy)Re(CO)_3_Cl. Excitation spectra for a pure, 60(±5) nm-thick, film of PVK (solid line with squares) detected at 410 nm, PL of the same PVK film on glass substrate excited at 350 nm (dashed line with squares), excitation spectra for a pure, 40(±3) nm-thick, film of (2,2'-bipy)Re(CO)_3_Cl (solid line with circles) detected at 540 nm, and PL of the same film on glass substrate excited at 350 nm (dashed line with circles).

Figure 1 shows the excitation and PL spectra of PVK and (2,2'-bipy)Re(CO)_3_Cl films on glass substrates. The excitation spectra for a pure, 60(±5) nm-thick, film of PVK (solid line with squares) shows the signal at 410 nm of the PL when the excitation wavelength is scanned from 250 to 380 nm. The spectra shows clear separated maxima at approximately 340 and 300 nm, with a distinct shoulder at 330 nm. The dashed line with squares give the corresponding PL of the same PVK film on a glass substrate excited at 350 nm with a maximum at 410 nm.

Additionally, Figure 1 shows the excitation spectra for a pure, 40(±3) nm-thick, film of (2,2'-bipy)Re(CO)_3_Cl (solid line with circles), which gives the detector signal at 540 nm of the PL when the excitation wavelength is scanned from 300 to 520 nm. The spectra show typical strong electronic ^1^ππ* intra-ligand transitions of the diimine ligands in the higher energetic region, that is, at wavelengths shorter than 330 nm, and metal-to-ligand charge-transfer (MLCT) signatures at lower energies, with a maximum at about 370 nm.[15, 16] The dashed line with circles shows the PL of the same (2,2'-bipy)Re(CO)_3_Cl film on a glass substrate excited at 350 nm. The broad emission of the ^3^MLCT to the ground state of compound (2,2'-bipy)Re(CO)_3_Cl is covering a typical wide spectral region including blue, green, and yellow light with its maximum at around 540 nm. The excitation spectra are normalized to the spectra of the excitation lamp (photons per wavelength), measured by an internal, calibrated silicon diode.

**Figure 2:**
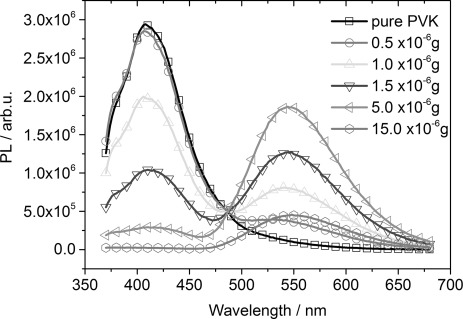
PL quenching experiments with PVK as a donor polymer and different amounts of (2,2'-bipy)Re(CO)_3_Cl catalyst as the quenching material on a glass/ITO substrate. The material was drop-cast from a solution, and the spectra were corrected to their corresponding absorption. The amount of PVK in all samples was 5(±0.2)×10^−4^ g.

Figure 2 shows PL quenching experiments with PVK as the donor polymer and (2,2'-bipy)Re(CO)_3_Cl catalyst as the quenching material on a glass/indium tin oxide (ITO) substrate. The materials were drop-cast from dichloromethane (DCM) solution. The PL spectra were corrected by the absorption of each film and by taking an average value of 10 % for the contribution of light scattering into account. The amount of PVK in all samples was 5(±0.2)×10^−4^ g. In Figure 2, the solid line with black squares shows the PL of a pure, approximately 1.8(±0.1) μm-thick, PVK film on a glass substrate that was excited at 350 nm. It shows a clear PL maximum at about 410 nm. As soon as some catalyst material is present, the PL of the PVK polymer is reduced and the typical catalyst PL, with a maximum at 553 nm, appears. At a quencher amount of approximately 15(±0.5)×10^−6^ g (about 3 % of the total material in the film), the PL signal of the PVK donor polymer disappeared completely and only the PL signal of the acceptor catalyst at 550 nm is present.

**Figure 3:**
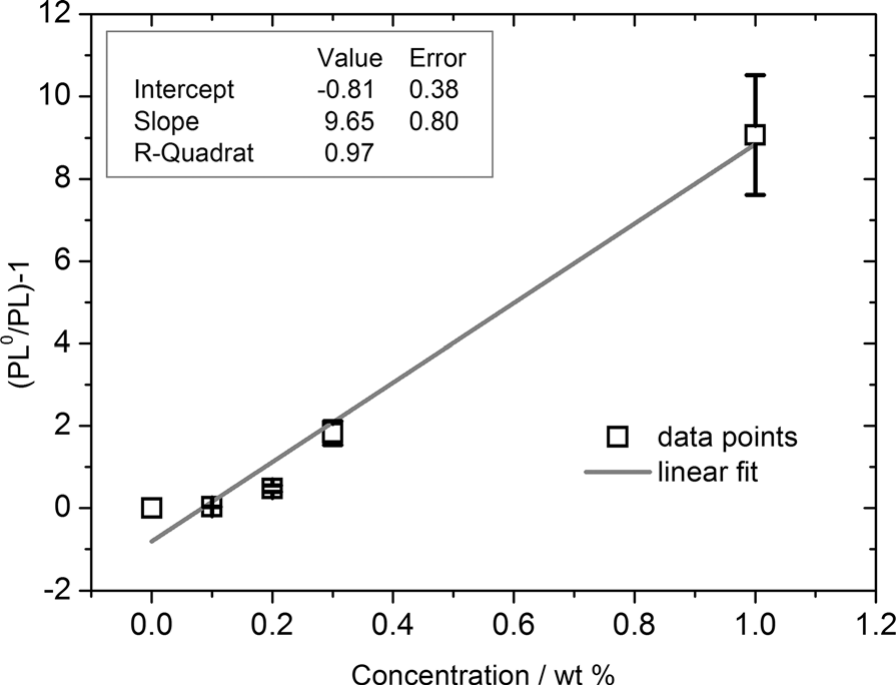
Stern–Volmer plot of the data represented in Figure 2, where *PL*^0^ is the PL intensity of the pure PVK without quencher and *PL* is the PL when a certain concentration of the quencher is added. The plot of (*PL*^0^/*PL*)−1 versus the quencher concentration gives a straight line.

Figure 3 shows the Stern–Volmer plot of the data represented in the Figure 2, where *PL*^0^ is the PL intensity of the pure PVK polymer without quencher at its maximum of approximately 410 nm and *PL* is the PL intensity when a certain concentration of the quencher is added. The last curve from Figure 3, with a quencher amount of 15(±0.5)×10^−6^ g, is not represented, as the PL signal is almost negligible and, hence, difficult to evaluate. Furthermore, the inhomogeneity of the film increases with increasing rhenium catalyst concentration, leading to the lower PL signal of the catalyst acceptor at high concentrations. According to the Stern–Volmer equation [Eq. (1)], a plot of (*PL*^0^/*PL*)−1 versus the quencher concentration [*Q*] should give a straight line, which is observed for this system to a good degree (with an *R*-squared value of 0.97).[17]


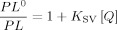
(1)

In Equation (1), *K*_SV_ is the Stern–Volmer constant and [*Q*] is the quencher concentration. Evaluating Equation (1) for the data represented in Figure 3 results in a *K*_SV_ value of 9.7(±1.5)×10^2^ g_Donor_ g^−1^_Quencher_. In the case of dynamic quenching, for example, in Förster resonance energy or Dexter-type electron transfer, *K*_SV_ is the product of the true quenching constant *k*_q_ and the excited state lifetime *τ* in the absence of the quencher material, according to Equation (2).


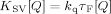
(2)

The fluorescence lifetime of PVK (*τ*_F_) has been reported to be 5.34(±0.03) ns.[18] Evaluating Equation (2) for a *K*_SV_ of 9.7(±1.5)×10^2^ g_Donor_ g^−1^_Quencher_ and *τ*_F_ equal to 5.34(±0.03) ns results in a *k*_q_ value of 1.8(±0.3)×10^11^ g_Donor_ g^−1^_Quencher_ s^−1^. This *k*_q_ value is then interpreted as the bimolecular reaction rate constant for the elementary reaction of the excited state with the quencher material.

**Figure 4:**
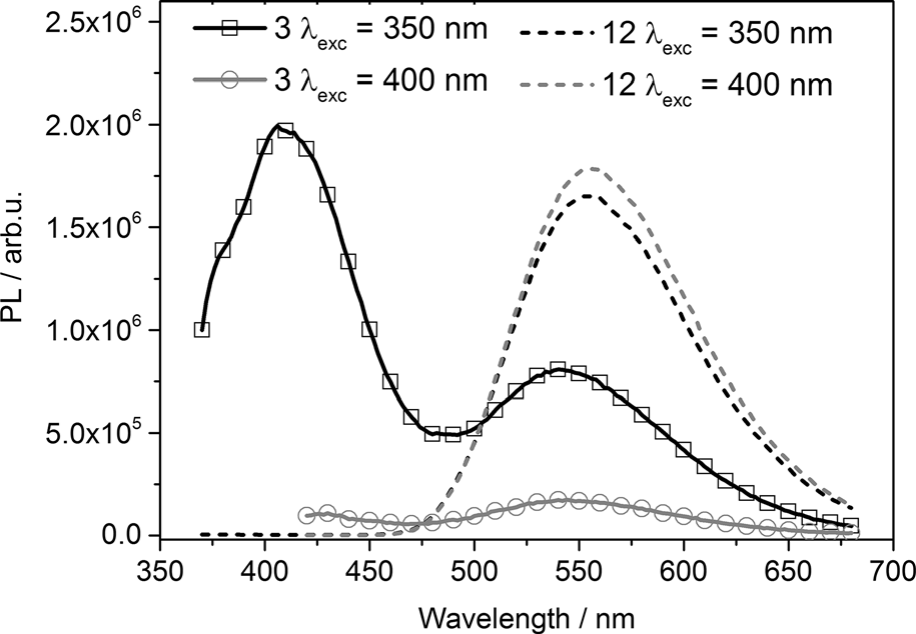
PL spectra with PVK as the donor polymer and 1(±0.1)×10^−6^ g (solid lines) and 6(±0.4)×10^−6^ g (dashed lines) of (2,2'-bipy)Re(CO)_3_Cl catalyst as the quenching material on a glass/ITO substrate. The material was drop-cast from a solution, and the spectra were corrected to their corresponding absorption. The amount of PVK in all samples was 5(±0.3)×10^−4^ g. The solid films were excited at two different wavelengths, namely 350 and 400 nm.

Figure 4 shows PL quenching experiments with PVK as the donor polymer and two different amounts of (2,2'-bipy)Re(CO)_3_Cl catalyst as the quenching material. The samples were drop-cast from DCM solution. The PL spectra were corrected by the absorption of each film and by taking an average value of 10 % for the contribution of light scattering into account. The PVK amount in all samples was 5(±0.3)×10^−4^ g. The solid films were excited at two different wavelength, namely 350 nm, which is the maximum of the PVK donor and relative minimum of the catalyst acceptor, and at 400 nm, which is close to the maximum of the ^3^MLCT catalyst acceptor absorption. The experiment was depicted for two different amounts of the catalyst acceptor, 1(±0.1)×10^−6^ g (solid lines) and 6(±0.4)×10^−6^ g (dashed lines). In the experiment with the lower amount, by exciting at the polymer absorption maximum at 350 nm, two luminescence peaks are observed, one characteristic of the PVK donor polymer at about 400 nm and the other at about 550 nm, characteristic of the luminescence of the catalyst acceptor (2,2'-bipy)Re(CO)_3_Cl. When the sample was excited at 400 nm, namely at the ^3^MLCT maximum of the acceptor (2,2'-bipy)Re(CO)_3_Cl, no PL signal of the donor polymer was present and only a weak PL signal of the catalyst material could be observed. At high catalyst quencher amounts (dashed lines), independent of the excitation wavelength, no PL signal of the PVK donor was present anymore; however, for both wavelengths, a strong PL signal of the acceptor catalyst (2,2'-bipy)Re(CO)_3_Cl was observed. Additionally, ESR experiments were carried out to study this system in bulk, in solid phase mixtures of donor and acceptor, and at a donor–acceptor solid–liquid interface. Following the argumentation of these results, together with the fact that, for this system, no light-induced ESR signal could be observed, which would be expected for unpaired electrons, owing to a charge transfer, the process seems to follow a Förster- and/or Dexter-type energy transfer from PVK to (2,2'-bipy)Re(CO)_3_Cl, with only a minor contribution of charge transfer involved.

### 2.2. PL Quenching in Acetonitrile Solution

Bulk solid donor–acceptor mixtures cannot currently be used for the application of CO_2_ reduction to a synthetic fuel with photoinduced energy transfer from PVK as a donor material to (2,2'-bipy)Re(CO)_3_Cl as a catalyst acceptor, unless a good way is found to efficiently provide the CO_2_ substrate and the electrons. In typical applications reported in the literature, the CO_2_ substrate is provided by purging an organic solvent [DCM, acetonitrile (ACN), etc.] until gas saturation and by dissolving the catalyst material, forming a homogenous solution.[19, 20] For PL quenching in ACN solution, the PVK donor was put at one side of the optical quartz cuvette and the excitation light was introduced from the PVK side to filter out 350 nm light and to avoid the direct excitation of the Re complex. As the concentration of the rhenium complex was sufficiently high, it does not need to diffuse to the surface of the PVK membrane in order to receive the energy; hence, the quenching efficiency is a function of the surface concentration of the rhenium complex, so that it exactly follows the Stern–Volmer relationship.

**Figure 5:**
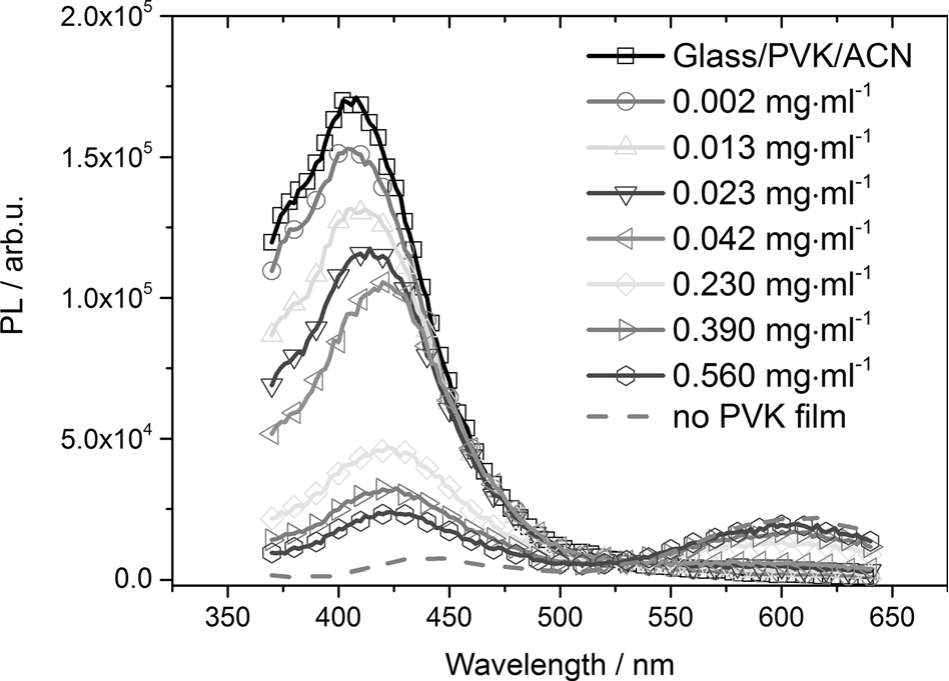
PL quenching experiments with a solid, 10(±0.7) nm-thick, film of PVK on ITO/glass substrate as the donor polymer and different concentrations of (2,2'-bipy)Re(CO)_3_Cl catalyst as the quenching acceptor added to the ACN solution.

Figure 5 shows PL quenching experiments with a solid, 10(±0.7) nm-thick, film of PVK on a ITO/glass substrate as the donor polymer, immersed into an ACN solution. The solid line with squares shows the PL signal of the pure PVK substrate in CAN, excited at 350 nm. In the subsequent spectra, the (2,2'bipy)Re(CO)_3_Cl catalyst was added as a quenching material into the ACN solution. With increasing concentration of the catalyst material, clear quenching is observed with a similar behavior as previously shown for the bulk, solid donor–acceptor mixtures. The catalyst PL with its maximum shifted slightly to around 600 nm appears, whereas the PVK luminescence with its maximum at about 410 nm vanishes almost completely once a catalyst concentration of 0.56(±0.02) mg mL^−2^ is reached (curve with circles). The shift of the catalyst PL compared to Figure 2 is most probably caused by the fact that it is now in solution. The gray dashed line shows the PL signal at the end of the quenching experiment when the PVK film was removed from the ACN solution. By looking at the spectra recorded in Figure 5, one has to take into account that only a few surface monolayers of donor and acceptor molecules get the chance to interact, and the bulk of the material in the solid phase and that dissolved in solution will not take part in the actual energy-transfer reaction. Following this argumentation, it is remarkable to observe a strong PL quenching effect in this solid–liquid donor–acceptor system.

**Figure 6:**
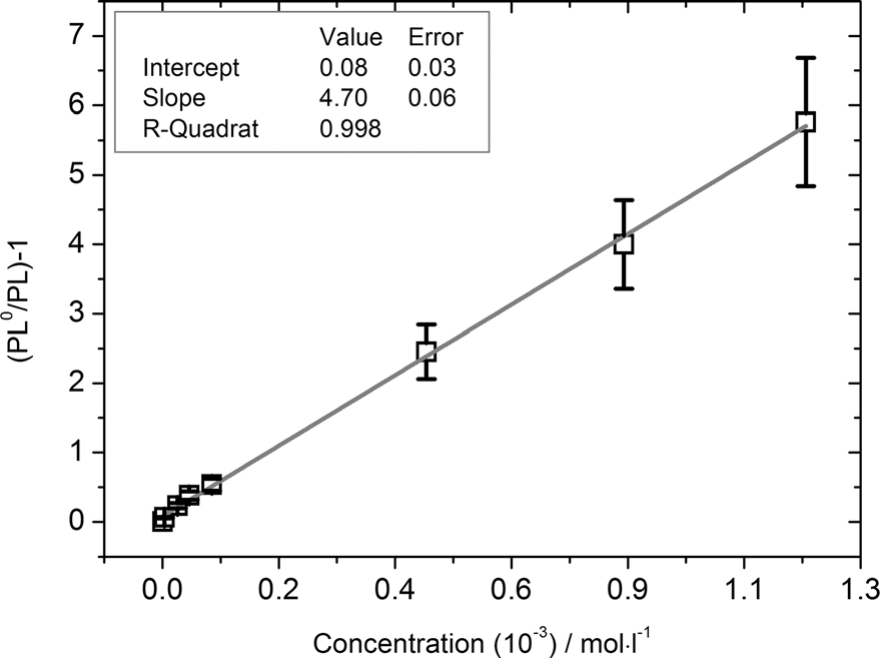
Stern–Volmer plot of the data represented in the Figure 4, where *PL*^0^ is the PL intensity of the pure PVK polymer film without quencher present, and *PL* is the PL when a certain concentration of the quencher is added to the ACN solution. The plot of (*PL*^0^/*PL*)−1 versus the quencher concentration gives a straight line.

In Figure 6, the data from Figure 4 is depicted in a Stern–Volmer plot, where *PL*^0^ is the PL intensity of the pure PVK polymer film without any quencher present, and *PL* is the PL when a certain concentration of the quencher is added into the ACN solution. The plot of (*PL*^0^/*PL*)−1 versus the quencher concentration gives an almost perfect straight line (*R*-squared value of 0.998). Evaluating Equation (1) for the data represented in Figure 6 results in a *K*_SV_ value of 4.7(±0.7)×10^3^ L mol^−1^. Evaluating again Equation (2) for a *K*_SV_ of 4.7(±0.7)×10^3^ L mol^−1^ and *τ*_F_ of 5.34(±0.03) ns results in a quenching or bimolecular reaction rate constant *k*_q_ of 8.8(±1.4)×10^11^ L mol^−1^ s^−1^.

## 3. Conclusions

In this work, to the best of our knowledge, PVK is used for the first time as a good and cheap absorber material in combination with the catalyst (2,2'-bipy)Re(CO)_3_Cl. The obtained results from PL quenching experiments and Stern–Volmer analysis show that PVK can act efficiently as a redox photosensitizer in combination with (2,2'-bipy)Re(CO)_3_Cl as a catalyst acceptor, leading to possible applications in photocatalytic CO_2_ reduction. This catalytic material is known to effectively reduce CO_2_ to CO, which might open new opportunities for the photochemical reduction of CO_2_.

## Experimental Section

### General Experimental Procedure

Unless otherwise stated, all chemicals and solvents were purchased from commercial suppliers in reagent–analytical grade quality and used directly as received, including DCM **(**EMSURE), ACS, ISO, Reag. Ph Eur, DCM for analysis (*M*=84.93 g mol^−1^), water-free ACN (Aldrich), water-free toluene (Aldrich), PVK (Aldrich; 368350–5G, secondary standard, *M*p 300 °C, *d*=1.2, polydispersity≈2, average *M*=35 000). Samples for PL quenching experiments were prepared either by drop-casting or spin-coating from solution onto glass or a glass/ITO substrate. The film thicknesses were measured on a Bruker DektakXT stylus profiling system, using a stylus-type radius of 2 μm, a stylus force of 5 mg, and a lateral resolution of 0.33 μm pt^−1^ over 1000 μm.

### (2,2'-Bipy)Re(CO)_3_Cl

The rhenium complex was prepared with slight modifications to literature methods.[21, 22] An equimolar amount of Re(CO)_5_Cl (0.304 g, 0.84 mmol) and 2,2'bipyridine (0.131 g, 0.84 mmol) was dissolved in hot toluene (50 mL). The reaction mixture was stirred under reflux for 2 h. Afterwards the solution was removed from the heat source and cooled down by an ice-salt mixture. The product precipitated from solution as a yellow powder and was filtered off. The powder was further dried at a rotary evaporator (5 mbar for 1 h). Pure product was isolated from the reaction with an overall yield of 92 %. ^1^H NMR (200 MHz, CD_3_CN): *δ*=7.62 (t, 2 H, bipy-H 5 and 5'), 8.18 (t, 2 H, bipy-H 4 and 4'), 8.41 (d, 2 H, bipy-H 6 and 6'), 9.00 ppm (d, 2 H, bipy-H 3 and 3').

### UV/Vis Spectroscopy

Wavelength scans were taken on a PerkinElmer Lambda 1050 double monochromator spectrometer (source-doubling mirror) between 400–700 nm in 2 nm steps, with a slit width of 2 nm and a detector response time of 0.2 s. Signal-to-noise was optimized by attenuating the reference beam with internal attenuators (automatic 2 and 3 A attenuation). For all spectra, auto-zero (100 and 0 %) correction scans were taken (baseline correction). The spectrometer was equipped with a deuterium (D_2_) lamp as the UV source and a tungsten lamp as the visible and near-infrared source. The energy was dispersed into specific wavelengths by using a reflective gratin. The source change was set to 320 nm. Photomultiplier R6872 and PMT were used for detection in the UV/Vis, a Peltier cooled InGaAs detector for use in the 800–2600 nm region, and a Peltier cooled PbS detector for the range from 2500–3300 nm.

### PL Spectroscopy

PL spectra were recorded on a PTI QuantaMaster 400 Spectrofluorimeter with a continuous Xenon arc lamp (75 W) light source in the emission range 185–680 nm, a Czerny–Turner type excitation monochromator (throughput 65 % at 300 nm) with a focal length of 300 mm, a excitation grating with 1200 lines per mm (300 nm blaze), an emission grating with 200 lines per mm (400 nm blaze), and a multimode PTI PMT detector (model 914), with spectral response from 185 to 900 nm [quantum efficiency at 260 nm (Peak) 25.4 %]. For a schematic drawing of the experimental setup for PL quenching in ACN solution, see Figure S5 in the Supporting Information.

### ESR Measurements

ESR spectra were recorded on Bruker EMX X-band spectrometer (9.45 GHz) with a rectangular TE_102_ cavity and Oxford ESR910 continuous flow He cryostat operating between 4 and 300 K. For solutions containing polar solvents, highly absorbing microwave radiation were soaked into quartz capillaries and placed in ESR tubes, otherwise a small amount of the solutions was added directly into the ESR tube. The samples were measured in the dark and under illumination with a halogen lamp to check for light-induced species. The samples were measured at various power levels from 20 μW to 200 mW with a modulation amplitude of 1 Gauss.

## Supporting Information

As a service to our authors and readers, this journal provides supporting information supplied by the authors. Such materials are peer reviewed and may be re-organized for online delivery, but are not copy-edited or typeset. Technical support issues arising from supporting information (other than missing files) should be addressed to the authors.

miscellaneous_information

## References

[1] Kerr RA (2007). Science.

[2] Friedlingstein P (2008). Nature.

[3] Yu KMK, Curcic I, Gabriel J, Tsang SCE (2008). ChemSusChem.

[4] Balzani V, Credi A, Venturi M (2008). ChemSusChem.

[5] Kumar B, Llorente M, Froehlich J, Dang T, Sathrum A, Kubiak CP (2012). Annu. Rev. Phys. Chem.

[6] Spinner NS, Vega JA, Mustain WE (2012). Catal. Sci. Technol.

[7] Hawecker J, Lehn J, Ziessel R (1984). J. Chem. Soc. Chem. Commun.

[8] Hawecker J, Lehn J, Ziessel R (1986). Helv. Chim. Acta.

[9] Takeda H, Koike K, Inoue H, Ishitani O (2008). J. Am. Chem. Soc.

[10] Takeda H, Koike K, Morimoto T, Inumaru H, Ishitani O (2011). Adv. Inorg. Chem.

[11] Kelly TD, Matos GR

[12] Pérez-Gutiérrez E, Percino MJ, Chapela VM, Maldonado JL (2011). Thin Solid Films.

[13] Wang S, Yang S, Yang C, Li Z, Wang J, Ge W (2000). J. Phys. Chem. B.

[14] Cardona CM, Li W, Kaifer AE, Stockdale D, Bazan GC (2011). Adv. Mater.

[15] Ley KD, Schanze KS (1998). Coord. Chem. Rev.

[16] Portenkirchner E, Oppelt K, Knör G, Egbe DAM, Sariçiftçi NS (2013). Nanomater. Energy.

[17] Scandola F, Balzani V (1983). J. Chem. Educ.

[18] Boo BH, Ryu SY, Kang HS, Koh SG (2010). J. Korean Phys. Soc.

[19] Takeda H, Ishitani O (2010). Coord. Chem. Rev.

[20] Portenkirchner E, Oppelt K, Ulbricht C, Egbe DAM, Neugebauer H, Knör G, Sariciftci NS (2012). J. Organomet. Chem.

[21] Smieja JM, Kubiak CP (2010). Inorg. Chem.

[22] Worl LA, Duesing R, Chen P, Della Ciana L, Meyer TJ (1991). J. Chem. Soc. Dalton Trans.

