# Supplementary material for: Photoinduced Energy Transfer from Poly(N-vinylcarbazole) to Tricarbonylchloro-(2,2'-bipyridyl)rhenium(I)
Source: Chemphyschem. 2014 Aug 19;15(16):3634–8. doi: 10.1002/cphc.201402269 (PMC4501306; doi:10.1002/cphc.201402269)
Supplement: Supplementary file 1 — miscellaneous_information [file cphc0015-3634-sd1.pdf]

CHEMPHYSCHEM

Supporting Information

© Copyright Wiley-VCH Verlag GmbH & Co. KGaA, 69451 Weinheim, 2014

Photoinduced Energy Transfer from Poly(*N*-vinylcarbazole) to Tricarbonylchloro-(2,2′-bipyridyl)rhenium(I)

Engelbert Portenkirchner,* Dogukan Apaydin, Gottfried Aufischer, Marek Havlicek, Matthew White, Markus Clark Scharber, and Niyazi Serdar Sariciftci[a]

cphc_201402269_sm_miscellaneous_information.pdf

Supporting Information

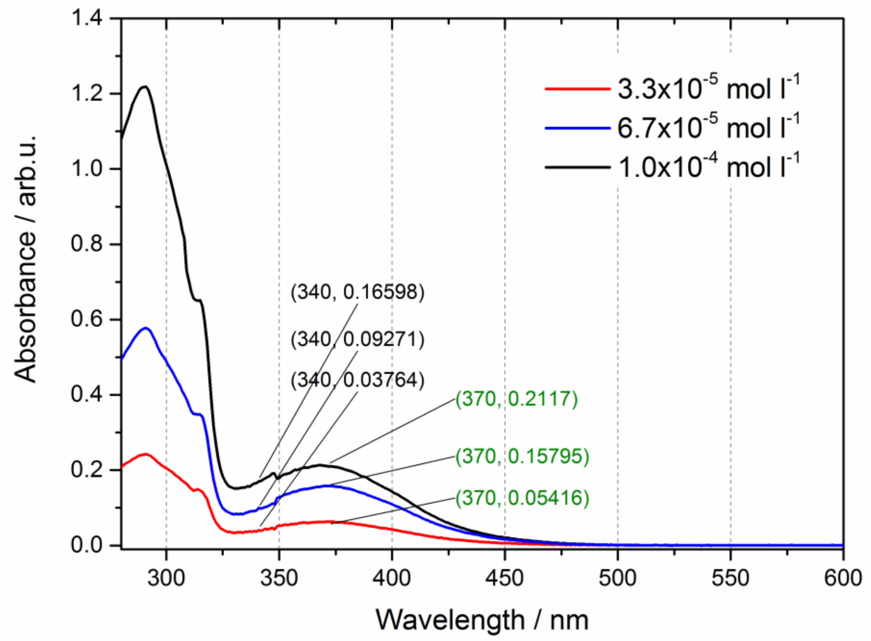

Figure S1. Absorption spectra of three different concentrations of fac-(2,2'bipy)Re$(CO)_3$Cl in acetonitrile solution.

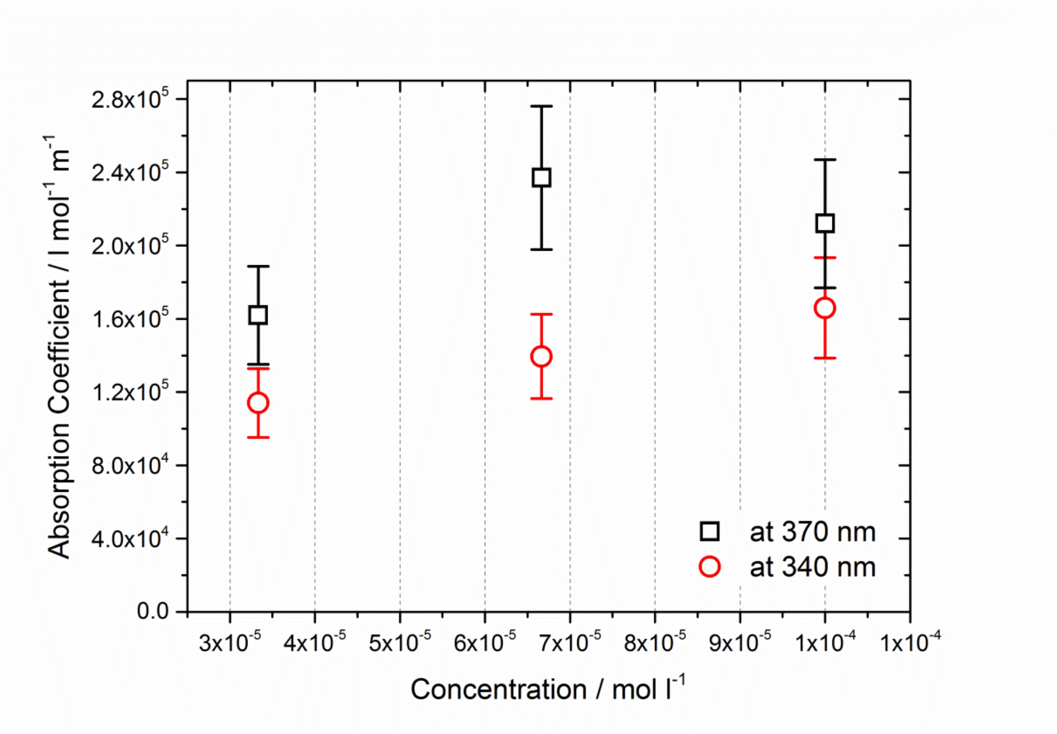

Figure S2 a. Calculated absorption coefficients out of the absorption spectra presented in Figures S1 for fac-(2,2'bipy)Re$(CO)_3$Cl at 340 nm (red circles) and 370 nm (black squares).

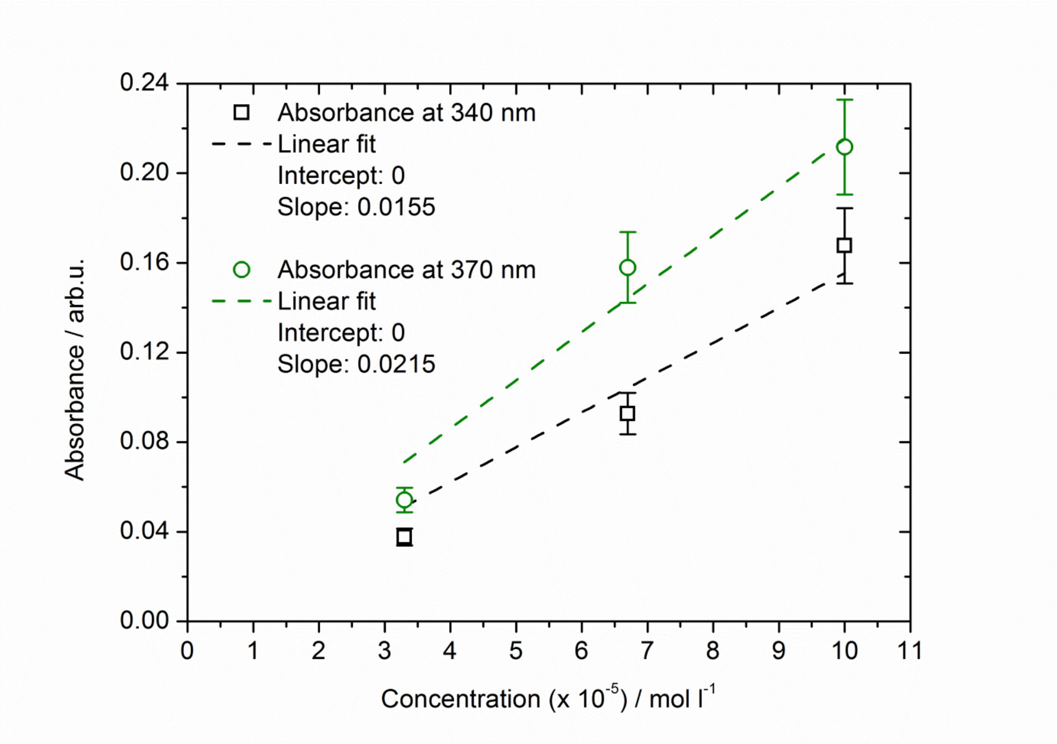

Figure S2 b. Plot of Absorbance vs. Concentration for the calculation of the absorption coefficients out of the absorption spectra presented in Figures S1 for fac-(2,2'bipy)Re$(CO)_3$Cl at 340 nm (black squares) and 370 nm (green circles).

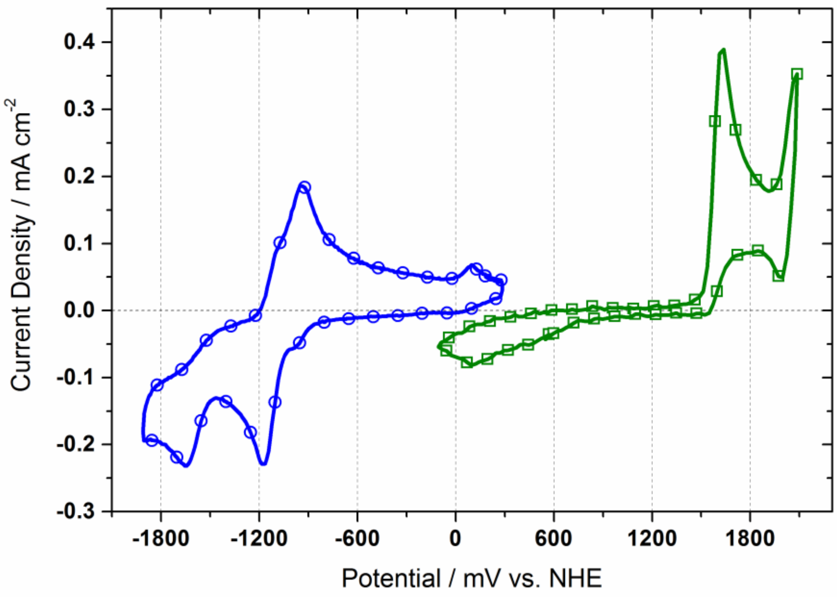

Figure S3. Cyclic voltammograms of fac-(2,2'bipy)Re(CO)$_3$Cl in nitrogen saturated electrolyte solution on the reductive side (blue line with circles) and oxidative side (green line with squares). Measurements are taken at a scan rate of 100 mVs^{-1} in acetonitrile with TBAPF6 (0:1 M), Pt working electrode, Pt counter electrode, and a concentration of 1mM.

Electrochemical experiments in Figure S3 were performed using a JAISSLE Potentiostat-Galvanostat IMP 88 PC. A one-compartment cell was used for cyclic voltammetry experiments with a Pt working electrode, a Pt counter electrode and a Ag/AgCl quasi reference electrode (QRE) calibrated with ferrocene/ferrocenium (Fc/Fc$^+$) as an internal reference. The half-wave potential ($E_{1/2}$) for Fc/Fc$^+$ was measured at 351 mV vs. QRE. For the recalculation to NHE potential the $E_{1/2}$ for Fc/Fc$^+$ vs. NHE was taken at 640 mV as suggested by Bazan *et al.*[1]

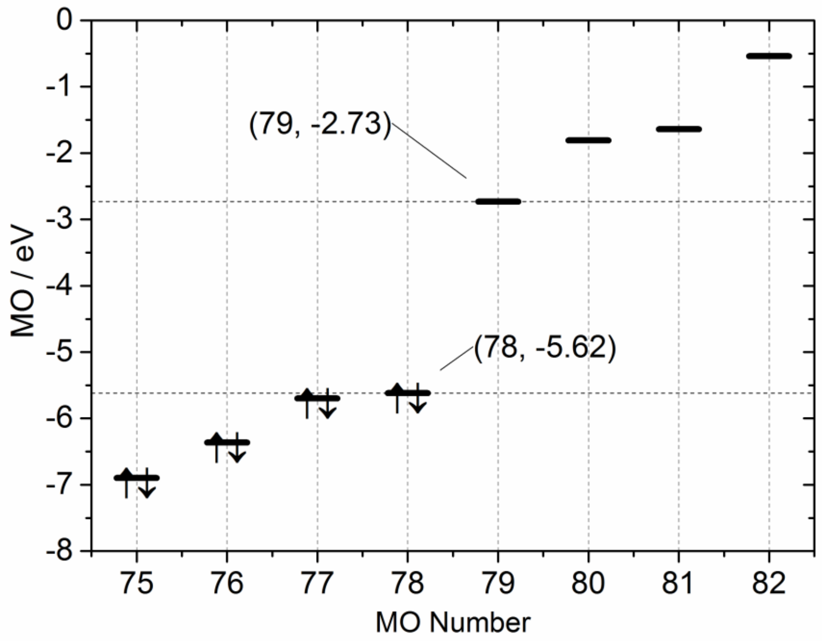

Figure S4. Molecular orbital energy levels of fac-Re(2,2'-bipyridyl)(CO)3Cl calculated by DFT for the frontier orbitals including HOMO-LUMO gap.

The supplementary Figure S4 shows the energy levels of the last four occupied and first four unoccupied molecular orbital (MO) of the novel compound (2,2'-bipyridyl)Re$(CO)_3$Cl obtained from theoretical calculations at the DFT level. The method used was similar to the one previously reported in reference nr [2]. The calculations were carried out with the Gaussian09.[3] All quantum-chemical calculations were carried out using a density functional theory (DFT) based method with the hybrid B3LYP functional.[4,5] The 6-31G(d) basis set was used through the calculations,[6,7] whereas for the complexed rhenium metal the LanL2DZ basis set[8] was applied. The obtained geometries were verified to correspond to a real minimum by establishing an absence of imaginary IR frequencies.

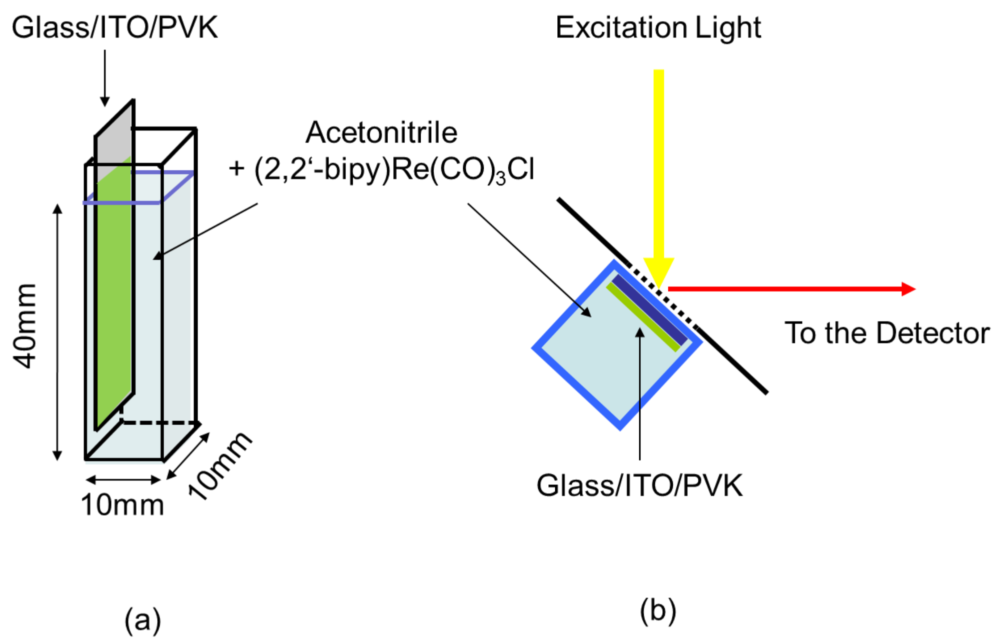

Figure S5. Experimental setup for photoluminescence quenching in ACN solution with PVK as donor material on glass/ITO substrate to (2,2'-bipy)Re(CO)3Cl as catalyst acceptor for bulk/solid and bulk/liquid donor-acceptor mixtures. (a) side view and (b) top view.

References:

1. Cardona, C. M., Li, W., Kaifer, A. E., Stockdale, D. & Bazan, G. C. Electrochemical considerations for determining absolute frontier orbital energy levels of conjugated polymers for solar cell applications. *Adv. Mater.* **23,** 2367–71 (2011).

2. Oppelt, K. *et al.* Luminescence and spectroscopic studies of organometallic rhodium and rhenium multichromophore systems carrying polypyridyl acceptor sites and phenylethynyl antenna subunits. *J. Organomet. Chem.* **696,** 2252–2258 (2011).

3. M. J. Frisch, G. W. Trucks, H. B. Schlegel, G. E. Scuseria, M. A. Robb, J. R. Cheeseman, G. Scalmani, V. Barone, B. Mennucci, G. A. Petersson, H. Nakatsuji, M. Caricato, X. Li, H. P. Hratchian, A. F. Izmaylov, J. Bloino, G. Zheng, J. L. Sonnenberg, M. Had, and D. J. F. Gaussian. (2009).

4. Becke, A. D. Density-functional exchange-energy approximation with correct asymptotic behavior. *Phys. Rev. A* **38,** 3098–3100 (1988).

5. Lee, C., Yang, W. & Parr, R. G. Development of the Colle-Salvetti correlation-energy formula into a functional of the electron density. *Phys. Rev. B* **37,** 785–789 (1988).

6. Hehre, W. J. Self-Consistent Molecular Orbital Methods. XIV. An Extended Gaussian-Type Basis for Molecular Orbital Studies of Organic Molecules. Inclusion of Second Row Elements. *J. Chem. Phys.* **56,** 5255 (1972).

7. Rassolov, V. a., Ratner, M. a., Pople, J. a., Redfern, P. C. & Curtiss, L. a. 6-31G* basis set for third-row atoms. *J. Comput. Chem.* **22,** 976–984 (2001).

8. Hay, P. J. & Wadt, W. R. Ab initio effective core potentials for molecular calculations. Potentials for the transition metal atoms Sc to Hg. *J. Chem. Phys.* **82,** 270 (1985).
